# Effects of Urban Landscape and Sociodemographic Characteristics on Heat-Related Health Using Emergency Medical Service Incidents

**DOI:** 10.3390/ijerph19031287

**Published:** 2022-01-24

**Authors:** Kanghyun Lee, Robert D. Brown

**Affiliations:** 1 Division of Landscape Architecture, College of Architecture, University of Oklahoma, Norman, OK 73019, USA; 2 Department of Landscape Architecture and Urban Planning, College of Architecture, Texas A&M University, College Station, TX 77843, USA; robert.brown@tamu.edu

**Keywords:** climate change, heat-related health, urban landscape characteristics, heat vulnerability

## Abstract

It is well known that extremely hot weather causes heat-related health issues. Health problems, especially in urban areas, are becoming increasingly important due to urban heat island effect. Understanding the impact of neighborhood characteristics is important for research into the relationship between thermal environment and human health. The objectives of this study were to explore the urban landscape and sociodemographic characteristics affecting heat-related health and identify spatial inequalities for vulnerable groups. A total of 27,807 heat-related EMS incidents were used at the census block group level (N = 285). We used land cover database and Landsat satellite images for urban landscape variables and used 2019 U.S. Census data for sociodemographic variables. Negative binomial regression was used to identify the neighborhood variables associated with the heat-related EMS incidents in each block group. Heat-related health has been alleviated in block groups with high green areas. However, the negative effects of thermal environments on human health were higher in areas with a high percentage of impervious surface, over 65 years, non-white people, no high school diploma, or unemployment. The results indicate that heat-related health problems can be addressed through prevention strategies for block group variables. Local intervention efforts to solve health issues should be targeted at more vulnerable areas and groups.

## 1. Introduction

It is well established that climate change is causing many areas of the world to experience an increase in frequency and intensity on extremely hot days [1]. Besides, more than half of the world population now lives in cities [2], and in counties like the United States and Canada, more than 80% of the population lives in urban areas [3]. As cities grow to accommodate the increasing population, they are often inadvertently built in such a way as to create an urban climate where the ambient temperature is higher than the prevailing conditions, a phenomenon known as the Urban Heat Island (UHI) [4].

Adverse effects of extremely hot days and high ambient temperatures caused by the two major trends on human health are well established [5,6,7]. Prolonged exposure to extremely high ambient temperatures can lead to increased heat-related morbidity [1]. In the United States, exposure to extreme heat from 2004 to 2018 resulted in a total of 10,527 deaths, with about 90% of deaths in hot weather from May to September [8]. Also, more than 30,000 deaths have been caused by heat waves during summer of 2003 in Western Europe, surpassing 739 heat-related deaths in the 1995 heat wave in Chicago, Illinois [9]. Due to global climate change, hot weather will become more intense, more frequent, and last longer in the late 21st century, and its impact on health will be more severe [10].

Previous studies have shown that urban landscape characteristics such as vegetation, surface, water bodies, and urban structure play an important role in mitigating the thermal environment [2,11,12,13]. These urban features have positive effects on heat-related health and have been used as essential factors for heat vulnerability assessment [14,15]. Sociodemographic characteristics are also a crucial part to be considered to address heat-related health problems [16,17]. Most heat-related health problems occur in urban areas because more than half of the world’s population lives in urban areas [18] and urban areas are more vulnerable to thermal environments due to higher temperatures than surrounding areas [19]. According to Lloyd-Sherlock [20], heat-related health issues are more pronounced in vulnerable groups such as the elderly, which has emerged as a major demographic trend. Also, various age groups, including infants, children, and the elderly are most vulnerable to heat-related death because they are more sensitive to excessive heat stress [21]. Heat vulnerability index (HVI) can be used to identify the effects of neighborhood characteristics such as vulnerable area or groups on heat-related health. HVI describes statistical and spatial patterns of heat vulnerability and consists of spatial explicit indices of exposure, sensitivity, and adaptability to heat [22]. HVI also can be used to construct vulnerability maps to determine areas requiring heat-related mitigation policies for extreme heat days [17,23].

The sociodemographic characteristics at the neighborhood level sometimes show different results depending on the internal correlation of variables. Some studies found that heat-related mortality increase with low socioeconomic status, such as education levels and buildings condition [16,24], but other studies have not confirmed any significant results [25,26]. These different results of the relationship between neighborhood characteristics and human health according to conditions such as population and location mean that the thermal vulnerability associated with social property is controversial and difficult to reach a single clear conclusion. Several studies have been attempted to explore the effect of neighborhood characteristics on heat-related health to address heat-related urban problems. However, there is a lack of empirical research that considers the variability of the relationship according to various neighborhood conditions. Also, most studies on heat vulnerability also have limitations in that they do not have finer spatial resolution than census track or county levels.

Therefore, the goal of this study was to conduct empirical research to explore neighborhood effects on heat-related health using emergency medical service (EMS) incident data in Cincinnati, Ohio (OH), over a 5-year period (2016–2020) at the census block group level. There were two neighborhood categories: urban landscape and sociodemographic characteristics. Normal and extreme heat days were considered to analyze the impact of hot weather on human health. This study aimed to explore neighborhood factors that affect heat-related health, identify spatial inequalities in heat-related EMS incidents, find the most heat-vulnerable areas by mapping heat vulnerability, and provide a foundation of knowledge for local interventions.

## 2. Materials and Methods

### 2.1. Study Design

We used daily heat-related health for the warm season between June and September (2016–2020) in Cincinnati, OH, which is in the mid-latitudes at around 39° N 84° W. Cincinnati is classified as a humid subtropical climate zone (Cfa, Köppen climate classification) and demonstrates an increasing trend with more extreme hot and humid weather types [27]. During the study period, the mean daily high temperature of normal heat days, 95th, and 97.5th extreme heat days were 84, 91, and 95° F, respectively. While many heat-related studies have used census tract level as the unit of analysis [2,24,28], we used census block group level to get more relative samples and improve the accuracy of analysis. Census block groups are more socially homogeneous than census tracts level since they are subdivisions of census tract [17,23]; 285 census block groups in Cincinnati were considered with a total population of about 302,000 in 2019.

### 2.2. Heat-Related Health Data

Heat-related Emergency Medical Service (EMS) data was used as a proxy for heat-related health. EMS data from 2016 to 2020 was obtained from the City of Cincinnati. The data provides information on date, time, latitude/longitude coordinates, and incident type. According to the Privacy Laws, latitude/longitude coordinates have been randomly skewed to represent values within the same block area of an incident. The Medical Priority Dispatch System (MPDS) determinant code is a way of categorizing and prioritizing EMS incidents. MPDS code consists of 32 categories. As described in previous studies, subcategories of heat-related MPDS codes were used [1,2]: code 06 (Breathing Problems), code 09 (Cardiac or Respiratory Arrest & Death), code 10 (Chest Pain, Non-traumatic), code 18 (Headache), code 20 (Heat & Cold Exposure), code 28 (Stroke & Cerebrovascular Accident), and code 31 (Unconscious & Fainting). The total daily heat-related EMS incidents for each code were calculated as the sum of 24-h EMS incidents. Latitude/longitude coordinates of each heat-related EMS incident were geocoded and assigned to the block group using ArcGIS 10.7 to calculate the total number of daily EMS counts for each block group. 

### 2.3. Heat Exposure Assessment

Daily maximum air temperature data were obtained from the Cincinnati Municipal-Lunken, OH weather station. Weather conditions were divided into normal heat (NH) days and extreme heat (EH) days. We defined the extreme heat with daily maximum temperatures above 95th and 97.5th percentiles of the time-period [24,28,29], and extreme heat days were defined as the daily maximum temperature above each threshold. Heat wave can be defined using extreme heat days with minimum duration of at least two or three days [30].

### 2.4. Urban Landscape and Sociodemographic Characteristics

For urban landscape characteristics, previous studies dealing with the relationship between neighborhood characteristics and outdoor thermal environments mainly focused on four categories: vegetation, surfaces, water bodies, and urban structure as urban landscape characteristics [12,13]. This study mainly focused on the effects of outdoor environments on heat-related health. Thus, we considered these four factors as urban landscape characteristics without considering the impact of the indoor environment such as buildings and houses. We considered these four factors as urban landscape characteristics. We considered these four categories as urban landscape characteristics and used the related variables for each category. Land cover database in 2016 with 30 m resolution was obtained from the US Geological Survey (USGS) and classified into tree covers, grass areas, impervious surfaces, and water bodies. The percentage of each classification for each block group was calculated using the TIGER/Line Shapefile in ArcGIS 10.7. 

We used two variables for urban structure. Urban density should be regarded as a key concept in the description of urban spatial structures [31]. Density is the population density of a city and it not only accounts for a significant portion of the compact city-related literature, but is also used as an attribute for estimating urban compactness [32,33]. We calculated population density for each block group using 2019 American Community Survey (ACS) data from the U.S. Census. Also, urban spatial structure can be explained by the volume of the built-up area [34]. We calculated the normalized difference built-up index (NDBI) to measure the distribution of urban structure within the block groups using the band 5 Near-Infrared (0.85–0.88 µm) and 6 SWIR 1(1.57–1.65 µm) of Landsat 8 satellite images at a resolution of 30 m. We also calculated land surface temperature (LST) using the band 10 TIRS 1 (10.6–11.19 µm) of Landsat 8 satellite images on July 2020, which showed the highest average temperature during the target period, at a resolution of 100 m for each block group [35]. To calculate the average value of NDBI and LST, pixel scores were aggregated to each block group. 

For the sociodemographic characteristics, we used HVI-based variables suggested by Nayak, Shrestha [22] and Reid, O’neill [36]. We included (1) percentage of over 65 years of age, (2) percentage of over 65 years of age and living alone, (3) percentage of living alone, (4) percentage of race other than white, (5) percentage with less than a high school diploma, (6) percentage below the poverty line, (7) percentage that are not employed, and (8) percentage of houses built before 1939. 2019 ACS data from the U.S. Census were collected for each block group. Variables were geocoded and assigned to the corresponding block group using ArcGIS 10.7. 

### 2.5. Statistical Analysis

We analyzed how the potential relationship between neighborhood environments and heat-related health outcomes varies depending on the level of spatial characteristics. When the dependent variable is the frequency of occurrence of events, count models are typically used to analyze the results, and negative binomial regression is commonly used to count variables when the dependent variable shows the skewed distribution and over-dispersed counts [37]. The negative binomial regression was used to predict an odds ratio (OR) because the dependent variable, which is the daily count of heat-related EMS incidents in each block group, showed the skewed distribution and overdispersion. Heat-related EMS was divided into three categories including normal, 95th extreme, and 97.5th extreme heat days according to temperature and three models were evaluated for each category. 

The analysis of this study can be mainly divided into three steps. First, univariate analysis was conducted for each of the independent variables. Second, after confirming the multicollinearity of the variables, multivariate analysis was performed using only statistically significant independent variables with a significant value of *p*-value < 0.05. R Studio version 1.3 was used to perform statistical analysis. Third, the heat vulnerability (HVI) map, which represent the relative risk (RR) of morbidity associated with hot days, was created. The values of each variable were normalized to have a mean of 0 and a standard deviation of 1. Then, the normalized variables were classified into six groups and scored from 1 to 6 points for each classification; 1 represents a low vulnerability and 6 represents a high vulnerability. HVI was calculated by summing all the scores for each block group and mapped to visualize the results. 

To use the count variable in the model, we need to identify the area in which the counts were generated. Size of census block group affects the occurrence of EMS incidents because the size of block groups varies, and more incidents may occur in larger block group. To control these effects, block group size was included in the analysis as a control variable. Also, to control the statistical probability that more incidents can occur in block groups with a larger population, population data of each block group was used as an exposure variable. Negative binominal regression can be used to describe expected rates when the rate is a count data divided by a specific unit of exposure such as population, and exposure variable can be used to modify each observation from a count into a rate per area [38].

## 3. Results

### 3.1. Summary Statistics for Heat-Related EMS and Neighborhood Characteristics

**Figure 1:**
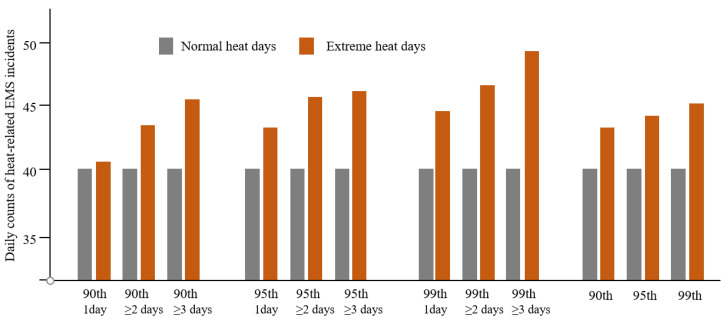
Comparison of daily number of heat-related EMS incidents between normal heat days and extreme heat days. An extreme heat day was defined using the intensity (90th, 95th, 99th) and duration (1 day, ≥2 days, ≥3 days).

There were a total of 29,270 heat-related EMS incidents during the warm season (2016–2020) in Cincinnati, OH. The daily count of heat-related EMS incidents ranged from 17 to 65, with an average of 39.9 (incidents/day). Figure 1 shows the comparison of the daily average number of heat-related EMS incidents during normal heat days and extreme heat days, including heat wave days and their daily average number of incidents. Overall, the number of EMS incidents on extreme heat days was higher than normal heat days, and it slightly increased with heat wave days. 

A total of 27,807 heat-related EMS data were used for the analysis, except for 1463 incidents that were missing location information. Of these, 66% (18,436) occurred on normal heat days, with 24% (6556) and 10% (2815), respectively, in 95th and 97.5th percentiles of extreme heat days. Table 1 shows descriptive statistics for all census block group variables, and most of the variables showed a wide range of variations. Overdispersion occurs when the observed variance is higher than the variance of a theoretical model [39]. The mean and standard deviation of EMS counts at 95th extreme heat days were 48.78 and 37.20. The relatively higher mean value was the same for other variables. According to the result of the overdispersion test using RStudio (qqc package), the p-values for the EMS counts at normal, 95th, and 97.5th extreme heat days were all less than 0.05. A *p*-value < 0.05 indicates spatially overdispersion and it means that the incidents were not equally distributed throughout the study areas because there are certain physical or sociological characteristics that affect this spatial overdistribution. Among the variables on urban landscape characteristics, water bodies showed the greatest variation, with the mean was 0.64%, and some block groups were more than 23% of water bodies. On average, 12% of the population were over 65 years of age, and in some block group, half the population was elderly. Similarly, the percentage of people living alone showed a high variability. Some block groups consisted almost entirely of people living alone, while others had relatively low rates.

Table 2 shows the correlations between block group variables. Relatively strong and positive correlations were observed between impervious surface, built-up area, and land surface temperature variables. Percent of tree cover was strongly and negatively correlated with these variables. Also, there were positive and strong correlations between variables, such as percent of unemployment, percent of below the poverty line, and percent of no high school diploma, which are related with social vulnerability. The results were used to identify the multicollinearity of variables for multivariate analysis.

### 3.2. Neighborhood Effects on Heat-Related EMS

**Figure 2:**
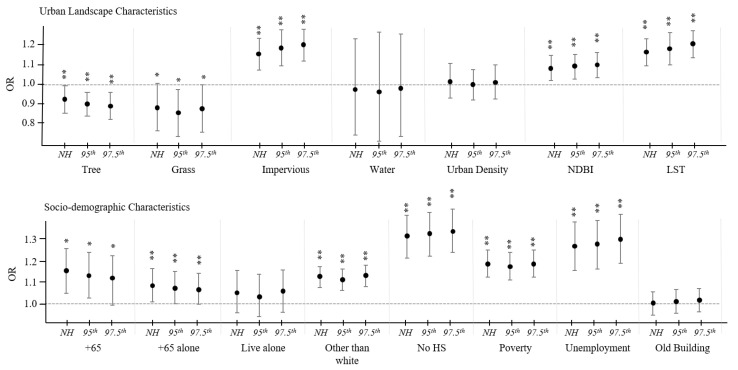
Odds ratios (ORs) and 95% confidence intervals for normal, 95th extreme, and 97.5th extreme heat days (** *p* < 0.01, * 0.01 ≤ *p* < 0.05).

Figure 2 shows how the relationship between neighborhood features and heat-related EMS varies depending on the block group variable for normal, 95th, and 97.5th extreme heat days. As a result, as the temperature rose, urban landscape variables such as impervious, NDBI, and LST affected the increase in heat-related EMS. Also, tree, green areas, impervious surfaces, NDBI, and LST variables were statistically significant with *p*-value < 0.05. We also found that the effect of percent of tree cover on the relationship between hot weather and heat-related health was stronger with higher temperatures, and the effects of percent of grass area were strongest in the 95th extreme heat days. As confirmed in correlation analysis, variables such as impervious surface, NDBI, and LST, which had high correlation with each other, showed similar results in a univariate analysis. Their impact on health became stronger as the weather got hotter. For the sociodemographic characteristics, variables such as percent of over 65 years of age, percent of over 65 years of age and living alone, percent of non-white, percent of no high school diploma, percent below the poverty line, and percent of unemployment were statistically significant. The impact of the elderly population and the elderly population living alone on heat-related EMS decreased as the weather got hotter, and the impact of variables such as high school degrees and unemployment rates increased as the temperature increased. Meanwhile, the percentage of buildings before 1939 and the percentage of people living alone were found to be insignificant in this analysis.

Table 3 shows the results of the multivariate analysis of the three models. Variables that were not significant (*p* > 0.05) in univariate analysis, such as percent of water area, population density, percent of living alone, and percent of buildings before 1939, were excluded from multivariate analysis. Also, NDBI and LST variables, which were highly correlated with the percent of impervious surface, were excluded from further analysis to avoid the multicollinearity issue. 

According to the results, the grass area had a greater effect on alleviating heat-related health on the 95th extreme heat day (OR = 0.839, 95% CI: 0.750–0.940, *p* < 0.01) than normal heat days (OR = 0.847, 95% CI: 0.755–0.951, *p* < 0.01). In contrast, the negative effects of impervious areas on heat-related health increased as temperatures increased from normal heat (OR = 1.120, 95% CI: 1.065–1.177, *p* < 0.01) to 95th (OR = 1.141, 95% CI: 1.087–1.198, *p* < 0.01) and 97th (OR = 1.157, 95% CI: 1.099–1.219, *p* < 0.01) extreme heat days. 

In sociodemographic variables, a high percentage of over 65 years of age showed a negative impact on heat-related health, and the relative risk decreased as the temperature increased from normal heat (OR = 1.320, 95% CI: 1.209–1.441, *p* < 0.01) to 95th (OR = 1.300, 95% CI: 1.194–1.415, *p* < 0.01) and 97th (OR = 1.277, 95% CI: 1.166–1.399, *p* < 0.01) extreme heat levels. This may be due to the tendency of the elderly to avoid their outdoor activities on hot days because the total volume of physical activities in the elderly is influenced by meteorological factors such as mean ambient temperature [40]. Percent below the poverty line was only significant on normally heat level (OR = 1.083, 95% CI: 0.987–1.189, *p* < 0.01) and percent of unemployment was only significant on 97.5th extreme heat level (OR = 1.141, 95% CI: 1.038–1.255, *p* < 0.01).

**Figure 3:**
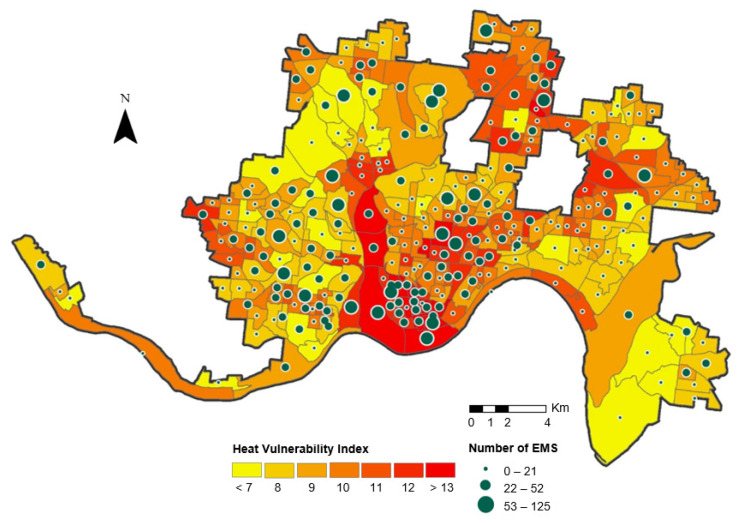
Heat vulnerability map and spatial distribution of heat-related EMS incidents for census block groups.

Figure 3 includes the heat vulnerability map and the number of heat-related EMS for each block group. Spatial inequalities in heat vulnerability and heat-related health through spatial analysis of the thermal environment can be confirmed on the map. Also, Spatial information for the vulnerable could be confirmed by utilizing the spatial inequality relationship between thermal vulnerability and heat-related EMS. The most vulnerable areas were the downtown of Cincinnati and several northern outskirts. This means that more heat-related EMS occur in areas with high HVI.

## 4. Discussion

This study explored the impact of neighborhood environments on heat-related human health using daily EMS data. Statistics and spatial analysis were used to investigate vulnerable areas to heat and to identify the spatial characteristics of these areas. The results found that heat-related accidents occur more often on extremely hot days than on normally hot days. The potential effects of block group variables, including individual and area-levels, was evaluated through the univariate approach that analyzes each variable individually. Also, the multivariate approach that is frequently applied in heat epidemiology was used to consider various variables together [16,24] and the results showed that the following variables have a statistically significant influence on heat-related health: percentage of grass area, percentage of impervious surfaces, percentage over 65 years of age, percentage of a race other than white, and percentage below the poverty line. Considering all variables comprehensively, the risk of heat-related EMS in the most heat-vulnerable areas has been investigated to increase significantly compared to relatively less vulnerable areas. 

The findings indicate that heat-related EMS incidents increase in block groups with less green space, consistent with the results from previous epidemiological studies exploring whether heat-related health associations vary by social and physical environmental characteristics. Urban green areas mitigate the urban heat island effect and play an important role in reducing heat stress during extreme heat days [41,42]. A study of U.S. Medicare participants showed that low green spaces cause hot temperatures, resulting in an increase in heat-related mortality [43]. Gronlund, Berrocal [16] conducted time-stratified case-crossover analysis using daily mortality in Michigan. They analyzed the modification effect of individual and ZIP code-level sociodemographic characteristics on extreme heat-related mortality among the elderly. Their results showed that green space is a significant modifier of the association between mortality and extreme heat. In the study of heat-related deaths in Phoenix from 2000 to 2008, the increase in green space showed a weak but significant association with the decrease in heat-related mortality probability in the target area as a separate variable [17]. These findings provide the basis for the main purpose of this study on the effect of green space, a representative element of the urban landscape, on heat-related health. In addition, the findings suggest that understanding the regional variations and characteristics of urban green spaces is crucial in heat vulnerability assessment along with the fact that green space is an essential factor to mitigate thermal environments and address heat-related problems.

This study found that the percentage of the population over 65 years was a statistically significant predictor of increased incident risk, consistent with the results of previous relevant studies. Benmarhnia, Deguen [44] found that heat vulnerability and heat-related health problems increased in the vulnerable groups, such as older adults aged over 65 and 75 and low individual socioeconomic status. Hendel, Azos-Diaz [45] also showed that the population most affected by the heat wave was those aged 65 or older, with night temperatures having the highest impact on heat wave mortality. As such, the elderly, along with vulnerable groups such as infants and young children, are more sensitive to heat stress and are most vulnerable to heat-related health [21].

Meanwhile, the percent of no high school diploma, percent of non-white, and percent of unemployment variables have shown significant results in this study. It means that these factors are related to heat vulnerability. They are also essential factors in social vulnerability assessment as well as heat vulnerability issues. Social vulnerability means that human health problems caused by external stress, including natural or human-caused disasters, can negatively affect local communities [46]. Flanagan, Hallisey [47] developed social vulnerability index (SVI) including four sections: socioeconomic status, household composition & disability, minority status & language, and housing & transportation. They defined the variables such as education, race, poverty, and unemployment as indicators for evaluating social vulnerability. Thus, the results of this study indicate that addressing heat-related problems for socially vulnerable groups is an essential challenge in urban areas along with the fact that socially vulnerable groups are also vulnerable to heat-related problems.

The percent of buildings before 1939 was not a significant predictor of increased risks in this study, while previous studies found significant results. One possible explanation is that, according to Gronlund [48], heat-related health problems were more serious in areas with a higher proportion of older buildings. However, he also found that housing conditions were not significant as an important predictor of heat vulnerability after controlling other characteristics such as the number of floors, how often left home in an average week, and number and type of fans. It means that the characteristics of buildings may vary depending on their geographical characteristics, which may affect the relationship between older buildings and human health.

This study provides insights to reduce heat-related problems, but there are several limitations. First, there was a lack of information on the air conditioning prevalence at block group level in this study. However, previous studies indicated that it is important not only to own air conditioners but also to have the financial resources to operate [49,50]. Thus, air conditioning prevalence can be captured at the local level through the poverty levels used in this study [16]. Nevertheless, the lack of detailed information on air conditioning prevalence is a significant limitation in heat vulnerability-related research, so further research will require a related analysis of block group levels. Second, this study only analyzed the modifying effects of the urban landscape and socio-demographic variables on heat-related morbidity at the census block group level, so did not necessarily consider individual associations. This is because individual-level data such as human health and demographic characteristics on a finer scale were not available. Third, the EMS data used in this study included latitude/longitude coordinates of each incident. According to privacy laws, however, coordinates were randomly distributed within the same urban block. It means that there is a limit to confirming the exact location information of heat-related EMS. Thus, further research is needed using data on smaller units. In addition, further research is needed to use more detailed spatial information. Fourth, this study used relatively coarse spatial resolution at the block group level. The distinction of green areas using satellite images with a resolution of 30 m cannot be accurately distinguished at the block group level. Spatial information about smaller spatial units may better explain the role of spatial properties of neighborhoods in health effects. Finally, this study used a cross-sectional approach that has limitations to sufficiently reflect causal inference between variables. Land cover database from 2016, American Community Survey (ACS) data from 2019, NDBI and LST Landsat satellite data from 2020 were used in this study. At the same time, our study covered a five-year period from 2016 to 2020. Other hard-to-control situations in the analysis beyond the scope of this study can affect the temporal and spatial distribution of incidents over the five-year period. In this regard, this study needs a premise that spatial patterns should remain largely unchanged to use data for different time periods. Thus, to identify causal relationships, longitudinal approaches should be considered in future research. 

## 5. Conclusions

The findings of this study suggest that multifaceted strategies for communities to create thermally comfortable and safe neighborhoods could play an important role in preventing heat-related incidents. A summary of the findings of this study is as follows. First, green areas among urban landscaping variables mitigated the effects of hot weather on heat-related health. In contrast, other variables such as impervious surface, NDBI, and LST showed a negative effect on heat-related health, indicating that NDBI and LST might be used as indicators to identify heat-vulnerable areas. Sociodemographic variables such as percent of over 65 years of age, percent of no high school diploma, percent of non-white, and percent of unemployment significantly affected the relationship between hot weather and health outcomes; in particular, socially vulnerable groups were more vulnerable to heat-related health. Second, we compared three models according to EMS categories, including normal and extreme heat-related data to find out the relationship between temperature changes. The finding is that the higher the temperature, the stronger the effect of block group variables on heat-related incidents. This means that the effects of hot weather on health outcomes can be mitigated through intervention in various neighborhood characteristics. Third, there were spatial inequalities in heat-related EMS incidents. Spatial patterns showed substantial variability between heat vulnerability, social vulnerability, and heat-related health. Urban centers and downtown areas showed higher heat vulnerability and more heat-related incidents, while suburban areas with high-income people, younger white populations, and greener environments were linked to reduced heat vulnerability and heat-related incidents. Finally, the heat vulnerability map of Cincinnati created in this study provides a foundation of knowledge for local interventions through information on where heat-related accidents occur what environmental factors affect. 

The results of this study provide urban planners, policy makers, sociologists, and public health experts with a cornerstone for solving social problems caused by the deteriorated thermal environment. Policy makers may use this map to make decisions on the selection of locations where additional medical facilities are needed. Urban planners can use the map to establish strategies to enhance the accessibility of rescue activities to vulnerable areas from heat-related accidents. In addition, the results can be used to establish interventions to public health experts to solve related public health problems. Public health experts can use the map and data to provide information on community or social care policies that currently exist or should be implemented in the future to vulnerable social groups such as the elderly in heat waves. These heat-related policies and suggestions can contribute to alleviating the UHI caused by climate change and urbanization, and furthermore, improving the relationship of climate change on human health.

## Figures and Tables

**Table 1:** Descriptive Statistics for census block group variables.

Category	Variable	Abbreviation	Mean	SD	Min	Max
Heat-related Morbidity	Normal heat days daily EMS	NH	48.78	37.20	2.50	275.50
	95th extreme heat days daily EMS	95th EH	23.00	17.03	0.00	125.00
	97.5th extreme heat days daily EMS	97.5th EH	9.88	8.51	0.00	54.00
Urban Landscape	Percent of tree cover	Tree	34.48	16.50	2.94	84.38
	Percent of grass area	Grass	21.33	6.91	1.07	52.28
	Percent of impervious surface	Imper	41.00	17.72	5.48	92.34
	Percent of water area	Water	0.64	2.68	0.00	23.33
	Population density (urban density)	Dense	9.27	5.97	0.23	32.41
	Average of NDBI * (built-up)	NDBI	−0.11	0.04	−0.20	0.02
	Average of LST **	LST	22.89	1.52	18.40	26.50
Socio-demographic	Percent of over 65 years of age	+65	12.93	8.76	0.68	47.53
	Percent of over 65 years of age and living alone	65+ alone	43.39	24.95	2.27	97.28
	Percent of living alone	Live alone	19.89	11.66	1.13	74.18
	Percent of non-white	Non white	51.47	29.19	3.32	97.95
	Percent of no high school diploma	No HS	13.45	11.39	0.23	61.29
	Percent below the poverty line	Poverty	26.04	19.45	0.18	86.23
	Percent of unemployment	Unemployment	10.05	10.00	0.48	64.41
	Percent of building before 1939	Old Building	43.01	25.06	1.05	95.83
Confounding Variables	Population	-	188	246	19	2781
	Size of block group	-	1085	553	155	4405

* Normalized difference built-up index. ** Land surface temperature (N = 285).

**Table 2:** Correlations for census block group variables.

Variable	Urban Landscape							Socio-Demographic							
	Tree	Grass	Imper	Water	Dense	LST	NDBI	+65	65+ Alone	Live Alone	Non White	No HS	Poverty	Unemployment	Old Building
Tree	1														
Grass	0.079	1													
Impervious	−0.856 **	−0.426 **	1												
Water	−0.059	−0.024	−0.160 **	1											
Density	−0.367 **	−0.137 *	0.493 **	−0.290 **	1										
LST	−0.897 **	−0.367 **	0.923 **	0.033	0.326 **	1									
NDBI	−0.906 **	−0.183 **	0.900 **	−0.062	0.458 **	0.897 **	1								
65+	0.044	0.186 **	−0.128 *	0.047	−0.310 **	−0.105	−0.062	1							
65+ alone	−0.138 *	−0.044	0.144 *	−0.060	0.144 *	0.147 *	0.149 *	0.091	1						
Live alone	−0.026	−0.114	0.111	−0.141 *	0.080	0.053	0.048	0.125 *	0.082	1					
Non white	−0.103	0.146 *	0.094	−0.204 **	0.059	0.163 **	0.153 **	0.036	0.100	0.003	1				
No HS	0.001	−0.056	−0.023	0.045	−0.114	0.088	−0.017	−0.033	0.053	−0.019	0.426 **	1			
Poverty	−0.094	−0.043	0.095	−0.055	0.098	0.159 **	0.080	−0.192 **	0.065	−0.048	0.453 **	0.514 **	1		
Unemployment	−0.093	−0.122 *	0.128 *	−0.023	0.135 *	0.181 **	0.092	−0.243 **	0.124 *	−0.054	0.519 **	0.638 **	0.600 **	1	
Old Building	−0.244 **	−0.268 **	0.318 **	0.045	0.105	0.274 **	0.254 **	−0.174 **	−0.101	−0.022	−0.173 **	−0.012	0.003	−0.044	1

** Correlation is significant at the 0.01 level. * Correlation is significant at the 0.05 level. (N = 285).

**Table 3:** Odds ratios (ORs) and 95% confidence intervals for heat-related EMS incidents during normal, 95th extreme, and 97.5th extreme heat days with multivariate analysis.

Category	Variable	Model 1 (Normal Heat)		Model 2 (EH 95th)		Model 3 (EH 97.5th)	
		OR	95% CI	OR	95% CI	OR	95% CI
Urban landscape	Grass area	0.847 **	0.755–0.951	0.839 **	0.750–0.940	0.861 **	0.765–0.972
	Impervious surface	1.120 **	1.065–1.177	1.141 **	1.087–1.198	1.157 **	1.099–1.219
Socio-demographic	Age > 65 years	1.320 **	1.209–1.441	1.300 **	1.194–1.415	1.277 **	1.166–1.399
	Age > 65 living alone	1.016	0.982–1.051	1.012	0.980–1.045	1.009	0.975–1.044
	Race other than white	1.070 **	1.033–1.109	1.069 **	1.033–1.107	1.081 **	1.042–1.121
	No HS diploma	1.084	1.021–1.152	1.130 **	1.035–1.234	1.119 *	1.018–1.229
	<Poverty line	1.083 **	0.987–1.189	1.056	0.996–1.120	1.044	0.982–1.111
	Unemployment	1.099	0.999–1.208	1.111	1.014–1.217	1.141 **	1.038–1.255
Confounding variable	Area	1.109 **	1.080–1.138	1.105 **	1.077–1.134	1.109 **	1.079–1.140
	Population	exposure	-	exposure	-	exposure	-

** Correlation is significant at the 0.01 level. * Correlation is significant at the 0.05 level. (N = 285).

## Data Availability

Not applicable.
